# Postoperative Mortality after Hip Fracture Surgery: A 3 Years Follow Up

**DOI:** 10.1371/journal.pone.0162097

**Published:** 2016-10-27

**Authors:** Oya Kilci, Canan Un, Ozlem Sacan, Mehmet Gamli, Semih Baskan, Mustafa Baydar, Bulent Ozkurt

**Affiliations:** 1 Department of Anesthesiology and Reanimation, Ankara Numune Training and Research Hospital, Ankara, Turkey; 2 Department of Orthopedics and Traumatology, Ankara Numune Training and Research Hospital, Ankara, Turkey; Georgia Regents University, College of Dental Medicine, UNITED STATES

## Abstract

**Background and Aims:**

To determine mortality rates and predisposing factors in patients operated for a hip fracture in a 3-year follow-up period.

**Methods:**

The study included patients who underwent primary surgery for a hip fracture.The inclusion criteria were traumatic, non-traumatic, osteoporotic and pathological hip fractures requiring surgery in all age groups and both genders. Patients with periprosthetic fractures or previous contralateral hip fracture surgery and patients who could not be contacted by telephone were excluded. At 36 months after surgery, evaluation was made using a structured telephone interview and a detailed examination of the hospital medical records, especially the documents written during anesthesia by the anesthesiologists and the documents written at the time of follow-up visits by the orthopaedic surgeons. A total of 124 cases were analyzed and 4 patients were excluded due to exclusion criteria. The collected data included demographics, type of fracture, co-morbidities, American Society of Anesthesiologists (ASA) scores, anesthesia techniques, operation type (intramedullary nailing or arthroplasty; cemented-noncemented), peroperative complications, refracture during the follow-up period, survival period and mortality causes.

**Results:**

The total 120 patients evaluated comprised 74 females(61.7%) and 46 males(38.3%) with a mean age of 76.9±12.8 years (range 23–95 years). The ASA scores were ASA I (0.8%), ASA II (21.7%), ASA III (53.3%) and ASA IV (24.2%). Mortality was seen in 44 patients (36.7%) and 76 patients (63.3%) survived during the 36-month follow-up period. Of the surviving patients, 59.1% were female and 40.9% were male.The survival period ranged between 1–1190 days. The cumulative mortality rate in the first, second and third years were 29.17%, 33.33% and 36.67% respectively. The factors associated with mortality were determined as increasing age, high ASA score, coronary artery disease, congestive heart failure, Alzheimer’s disease, Parkinson’s disease, malignancycementation and peroperative complications such as hypotension (p<0.05). Mortality was highest in the first month after fracture.

**Conclusion:**

The results of this study showed higher mortality rates in patients with high ASA scores due to associated co-morbidities such as congestive heart failure, malignancy and Alzheimer’s disease or Parkinson’s disease. The use of cemented prosthesis was also seen to significantly increase mortality whereas no effect was seen from the anesthesia technique used. Treatment of these patients with a multidiciplinary approach in an orthogeriatric ward is essential. There is a need for further studies concerning cemented vs. uncemented implant use and identification of the best anesthesia technique to decrease mortality rates in these patients.

## Introduction

Hip fractures have become an increasing health problem throughout the world in parallel with the global aging population**[1]**.The proportion of elderly people in the general population is rapidly increasing **[2–3].** With this shift in demographics, it is likely that as medical personnel, we will meet the elderly at least twice in their lifetime for surgical procedures **[3]**. Due to cognitive, motor, sensory, and autonomic nervous system changes, a reduction in the functional capacity of all organs and reduced reflexes with the aging process, the elderly become vulnerable to trauma and thereby to hip fractures **[2–5]**.

Hip fracture is associated with increased morbidity and mortality. Wagner reported that 25% of patients with a hip fracture become dependent on nursing, 50% recover to preoperative status and the remaining 25% die, usually within the first year postoperatively **[6].** Male gender, increasing age, cognitive function impairment and high ASA scores have been found to be significantly associated with mortality in these patients **[7]**.

However, some studies have also considered the mode of anesthesia and use of cemented prosthesis to be responsible for increased mortality**[8–10]**. A cemented prosthesis increases mobilization, decreases pain, and increases quality of life but may lead to death due to bone cement implantation syndrome. Orthopedic surgeons can improve patient survival rates through reduced usage of cemented implants. Anesthesiologists can also play an important role in preventing perioperative mortality by identifying the ‘‘best anesthesia” technique in hip fracture patients. There have been few studies concerning the effects of different anesthesia types on the mortality of these patients. Thus, these two issues remain as matter of debate.

Therefore, the present study was planned to determine the mortality rate and identify the risk factors of mortality in a 3-year follow-up period of patients who have undergone surgery for a hip fracture.The secondary aim of this retrospective analysis was to report the effects of anesthesia techniques and use of cemented vs. uncemented prosthesis on the mortality of these patients.

## Materials and Methods

This study was designed as a retrospective study, including patients who were admitted to the Orthopedics and Traumatology Department in our hospital and underwent primary surgery for a hip fracture. All traumatic, non-traumatic, osteoporotic and pathological hip fractures treated surgically in 2011 were retrospectively examined. No exclusion was made of gender or age. The exclusion criterias were periprosthetic fractures, previous contralateral hip fracture surgery, and patients who could not be contacted by telephone

The study was approved by the Ethics Committee of Ankara Numune Training and Research Hospital (171/2014) Anesthesia and hospital medical records were then obtained and data were collected on name-surname, IP number at hospital admission, age, type of fracture, type of surgery, type of prosthesis fixation(cemented or uncemented), type of anesthesia technique, perioperative complications such as hypotension, the presence of preoperative hypertension (HT), diabetus mellitus (DM), thyroid disease, coronary artery heart disease (CAHD), chronic obstructive pulmonary disease (COPD), chronic renal failue (CRF) or chronic renal disease (CRD), arrythmia, congestive heart failure (CHF), alcoholism and liver disease, Alzheimer’s disease, Parkinson’s disease, epilepsy, ASA scores (1: healthy, 2:mild systemic disease, 3: severe systemic disease, 4: severe systemic disease with a threat to life; functionally incapasitated, 5: not expected to live without operation), occupation at home or an institution, presence of infection and refracture in the follow-up period either periprosthetic or contralateral, mortality or survival and probable causes of death were recorded. The data were collected by the corresponding author and a co-author. A total of 4041 orthopedic operations were done in 2011 in our hospital and 124 of them were hip fracture surgery. Names, co-morbidities, peroperative complications and anesthesia types were transformed from the peroperative anesthesia record paper Information about type of fracture, type of surgery and fixation, occupation and infection were colleted from the Orthopedic surgeon and hospital software medical records. A structured telephone conversation (including introduction of the author to the person he/she is talking to; confirmation of the patient’s name; obtaining information about the patient’s health, and if the patient had died, the date of death and the reason if known) was held with the patient or close relatives/caregivers/nursing personnel to learn about mortality and its causes during the 3-years follow- up period. A total of 124 patient records were examined and 4 patients were excluded due to lack of contact by telephone conversation (change of number or died) in 1 patient and presence of previous hip fracture surgery in 3 patients.

### Statistical Analysis

Data analysis was performed using SPSS for Windows, version 11.5 **[**SPSS Inc., Chicago, IL, United States**]**. Descriptive statistics were shown as mean ± SD or number of cases and percentages, where applicable.

To determine the effect on overall survival, both demographic and clinical variables were evaluated with Univariate Cox Proportional Hazards Regression Analysis. Determination of the best predictor(s) which discriminated the surviving and exitus cases from each other was analyzed with the Multiple Cox Proportional Hazards Regression Backward LR method. Adjusted hazard ratios and 95% confidence intervals were also calculated for each independent factor. Any variable where the univariable test had a value of p<0.25 was accepted as a candidate for the multivariable model together with all the variables of known clinical importance **[11]**. A value of p<0.05 was considered statistically significant.

## Results

The final 120 patients evaluated comprised 74 females(61.7%) and 46 males (38.3%) (**Table 1**).

**Table 1:** Demographic and Clinical Features of the Patients.

Variables	n = 120
**Age (year)**	76.9±12.8
*Age Range(year)*	23–95
**Gender**	
*Female*	74 (61.7%)
*Male*	46 (38.3%)
ASA Score*	
*I*	1 (0.8%)
*II*	26 (21.7%)
*III*	64 (53.3%)
*IV*	29 (24.2%)
**Co-existing Diseases**	
HT*	74 (61.7%)
DM*	34 (28.3%)
*Thyroid Disease*	7 (5.8%)
*Coronary Artery Heart Disease*	40 (33.3%)
*Pacemaker*	2 (1.7%)
*Atrial Fibrillation*	12 (10.0%)
*Valvular Disease*	6 (5.0%)
*Congestive Heart Failure*	9 (7.5%)
*Chronic Obstructive Pulmonary Disease*	28 (23.3%)
*Chronic Renal Failure/Disease*	18 (15.0%)
*Cerebro Vascular Event*	19 (15.8%)
*Alzheimer*	17 (14.2%)
*Parkinson*	5 (4.2%)
*Alcoholism/Chronic Liver Disease*	16 (13.3%)
*Epilepsy*	4 (3.3%)
*Malignancy*	9 (7.5%)

*ASA(American Socirty of Anesthesiologists), HT(hypertension), DM(Diabetes mellitus)

The ASA score distribution of the patients was 0.8%, 21.7%, 53.3% and 24.2% for ASA I, ASA II, ASA III and ASA IV respectively (Tables **2 and 3**).

**Table 2:** Other Clinical Features of the Patients.

Variables	n = 120 (%)
**Anesthesia Type**	
• *General*	65 (54.2%)
• *Spinal*	23 (19.2%)
• CSE*	12 (10.0%)
• LSPNB*	20 (16.7%)
**Place of residence**	
• *House*	111 (92.5%)
• *Geriatric institution*	9 (7.5%)
**Type of Surgery**	
• *Partial Arthroplasty* • *Total Arthroplasty*	102 (85.0%)3 (2.5%)
• IMN Closed* • IMN Open*	8 (6.7%) 7 (5.8%)
**Type of Prosthesis Fixation**	
• *Cemented*	60 (50.0%)
• *Non-cemented*	60 (50.0%)
**Perioperative Complication** • *None* • *Hypotension* • *Embolism*	101 (84.2%) 18 (15.0%) 1 (0.8%)
**Side of Fracture**	
• *Right*	58 (48.3%)
• *Left*	62 (51.7%)
**Localisation of Fracture**	
• *Neck*	87 (72.5%)
• *Trochanteric*	33 (27.5%)
**Re-Fracture**	
• *None*	117 (97.5%)
• *Ipsilateral Periprosthetic*	2 (1.7%)
• *Contralateral Neck*	1 (0.8%)
**Status**	
• *Survival*	76 (63.3%)
• *Exitus*	44 (36.7%)
**Follow-up (days)**	993 (1–1190)

*CSE(Combined spino-epidural anesthesia), LSPNB(Lomber somatic and peripheral nerve block), IMN(Intramedullary nailing)

**Table 3:** The frequency distribution of anesthetic techniques according to ASA scores.

ASA*	GENERAL	SPINAL	CSE*	LSPNB*	SUM
I			1		1
II	16	4	4	2	26
III	34	15	7	8	64
IV	15	19		10	29
SUM	65	23	12	20	120

*ASA (American Society of Anesthesiologists), CSE (Combined spino-epidural anesthesia), LSPNB (Lomber somatic and peripheral nevre block)

With increasing ASA scores, mortality increased 3.305 times with each grade.It was a significant mortality risk both in univariant [HR = 3.305 (95%CI:2,040–5,353), p<0.001)]and multivariant analysis [HR = 2.147(95%CI:1.082–4.260), p:0.029](Table **4**).

**Table 4:** Effect of Demographic and Clinical Features of Patients on Overall Survival According to Univariate and Multiple Cox-Proportional Hazards Regression Analysis.

Variables	HR (95% CI)†	p-value†	HR (95% CI)‡	p-value‡
**Age**	1.087 (1.044–1.131)	**<0.001**	1.040 (0.988–1.094)	0.131
**Gender**				
*Female*	1.000	-	-	-
*Male*	1.106 (0.606–2.017)	0.743	-	-
ASA*	3.305 (2.040–5.353)	**<0.001**	2.147 (1.082–4.260)	**0.029**
**Co-existing Diseases**				
HT*	2.211 (1.117–4.378)	**0.023**	1.106 (0.517–2.367)	0.795
DM*	1.271 (0.674–2.397)	0.459	-	-
*Goitre*	0.772 (0.187–3.191)	0.721	-	-
CAHD*	2.271 (1.255–4.108)	**0.007**	2.090 (0.965–4.525)	0061
*Pacemaker*	0.048 (0.000–503.618)	0.521	-	-
AF*	2.291 (1.017–5.158)	**0.045**	1.854 (0.664–5.179)	0.239
*Valvular Disease*	1.613 (0.499–5.212)	0.424	-	-
CHF*	4.802 (2.117–10.894)	**<0.001**	4.648 (1.504–14.364)	**0.008**
COPD*	1.022 (0.505–2.068)	0.952	-	-
CRF/CRD*	1.323 (0.615–2.848)	0.474	-	-
CVE*	1.824 (0.900–3.695)	0.095	1.818 (0.784–4.216)	0.164
*Alzheimer*	3.206 (1.643–6.257)	**<0.001**	5.653 (2.176–14.683)	**<0.001**
*Parkinson*	6.063 (2.347–15.659)	**<0.001**	7.213 (2.145–24.252)	**<0.001**
*Alcoholism/Chronic Liver Disease*	0.421 (0.130–1.361)	0.149	0.562 (0137–2.307)	0.424
*Epilepsy*	0.688 (0.095–4.995)	0.711	-	-
*Malignancy*	2.246 (0.948–5.325)	0.066	8.036 (2.601–24.825)	**<0.001**
**Anesthesia Type**				
CSE*	1.000	-	1.000	-
*General*	2.162 (0.507–9.226)	0.298	3.240 (0.578–18.156)	0.181
*Spinal*	2.642 (0.507–9.226)	0.214	1.769 (0.285–10.977)	0.540
LSPNB*	4.342 (0.971–19.428)	0.055	5.570 (0.805–38.568)	0.082
**Place of Residence**				
*House*	1.000	-	-	-
*Geriatric Institution*	0.789 (0.244–2.548)	0.692	-	-
**Type of Surgery**				
IMN Open* IMN Closed*	1.000 1.781 (0.161–19.650)	- 0.637	- -	- -
*Partial Arthroplasty*	3.100 (0.426–22.547)	0.264	-	-
*Total arthroplasty*	-	0.975	-	-
**Per-op Complication**	1.925 (0.950–3.899)	0.069	2.812 (1.044–7.575)	0.041
**Fracture type**				
*Femoral neck*	1.000	-	1000	-
*Trochanteric*	1.666 (0.901–3.081)	0.104	1.361 (0.602–3.079)	0.459
**Prosthesis Fixation**				
*Non -cemented*	1.000	-	1.000	-
*Cemented*	2.076 (1.122–3.839)	**0.020**	2.624 (1.192–5.777)	**0.017**
**Re-fracture**	0.047 (0.000–92.964)	0431	-	-

*ASA(American society of Anesthesiologists), HT(hypertension), DM(Diabetes mellitus), CAHD(Coronary artery heart disease), AF(atrial fibrillation), CHF(congestive heart failure), COPD(chronic obstructive pulmonary disease), CRF/CRD(chronic renal failure/chronic renal disease), CVE(cerebral vascular events-thrombosis or hemorrhage), CSE(combined spino-epidural anesthesia), LSPNB(Lomber somatic block&peripheral nerve bolcks), IMN(intramedullary nailing)

HR: Hazards Ratio, CI: Confidence Interval

† Univariate Cox-Proportional Hazards Regression Analyses

‡ Multiple Cox-Proportional Hazards Regression Analysis after adjustment for all possible confounding factors.

The age range was between 23–95 years of age with an average of 76.9±12.8 years. With increasing age, mortality was statistically significantly high [HR = 1.087 (95%CI: 1.044–1.131), p<0.001)] (**Table 4**).

Hypertension, diabetus mellitus and coronary artery diseases were the most frequently seen comorbidities in the patients (**Table 1**).

The place of residence before the fracture was their own home in 111 patients (92.5%) and a geriatric institution in 9 patients (7.5%)(**Table 2**).

Of the total 120 patients, 87 (72.5%) had a femoral neck fracture and the remaining 33 (27.5%) had a trochanteric fracture of the femur. Mortality was determined as 32.2% (n: 28) in patients with femoral neck fracture and 48.5% (n: 16) in patients with trochanteric femoral fracture (p>0.05)(**Table 2**).The type of fracture was not determined to have a statistically significant effect on mortality (**Table 4)**.

The patients had surgery under general (54.2%), spinal (19.2%), combined spino-epidural (10%) anesthesia and peripheral nerve blocks (16.7%) (Tables **2 and 3**). The anesthesia technique had no statistically significant effect on survival in univariant statistical analysis (p>0.05) (**Table 4**).

The surgical techniques performed were hemiarthroplasty in 103 (87.5%), total arthroplasty in 3 (%2.5), and intramedullary nailing (IMN) in 15 (12.5%) cases (**Table 2**). There was no significant association between the surgical technique and mortality in univariant statistical analysis (p >0.05) (**Table 4**). Cementation for prosthesis fixation was used in 50% (n: 60) of the patients. Mortality was 46.7% in cemented and 26.7% in the uncemented patients and it was statistically significant both in univariant (p: 0.02) and multivariant analysis (p: 0.017) (**Table 4**). Two patients with uncemented prosthesis and seven patientswith cemented prosthesis died in the first postoperative week, especially two patients in the cemented group died within the first 24 hours postoperatively.

A total of 44 patients (36.67%) died within 3 years of the fracture. Of these 44 patients, 18 were males (40.9%) and 26 were females (59.1%). Of the 74 females and 46 males included in the study, mortality was determined as 35.14% in females and 39.13% in males. No significant difference was determined between the genders in respect of mortality rates(**Table 4**).

**Fig 1:**
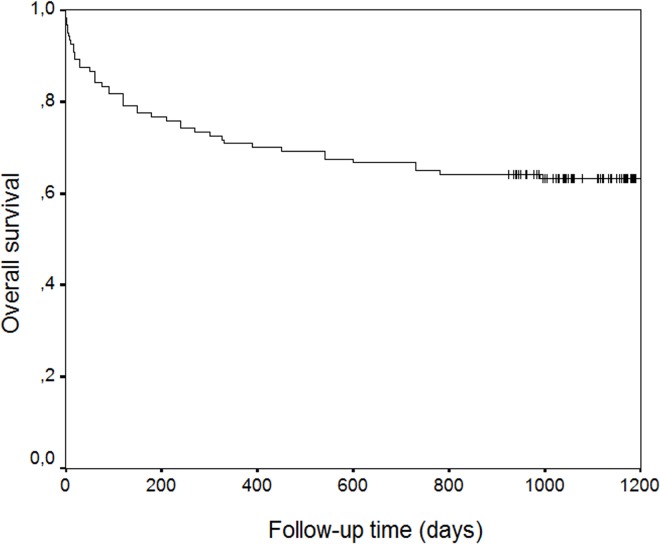
Kaplan Meier’s Overall Survival Rate Graphy.

In the 3-year period survival analysis of the total 120 patients, mortality was determined as 29.17% (35/120)in the first year, 33.33% (40/120) in two years and 36.67% (44/120) in three years (Fig 1).

**Fig 2:**
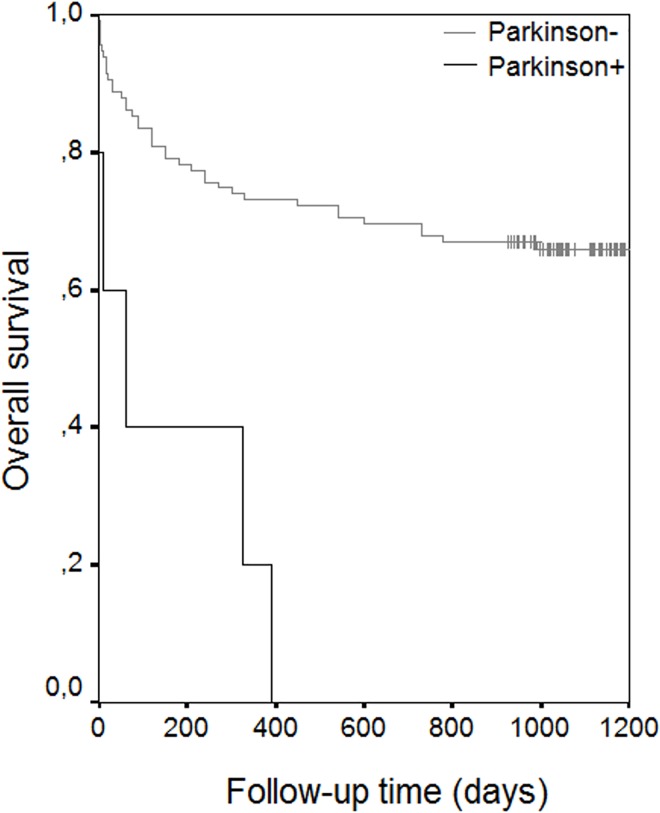
Overall Survival Rate Graphics of Patients with Alzheimer’s Disease.

**Fig 3:**
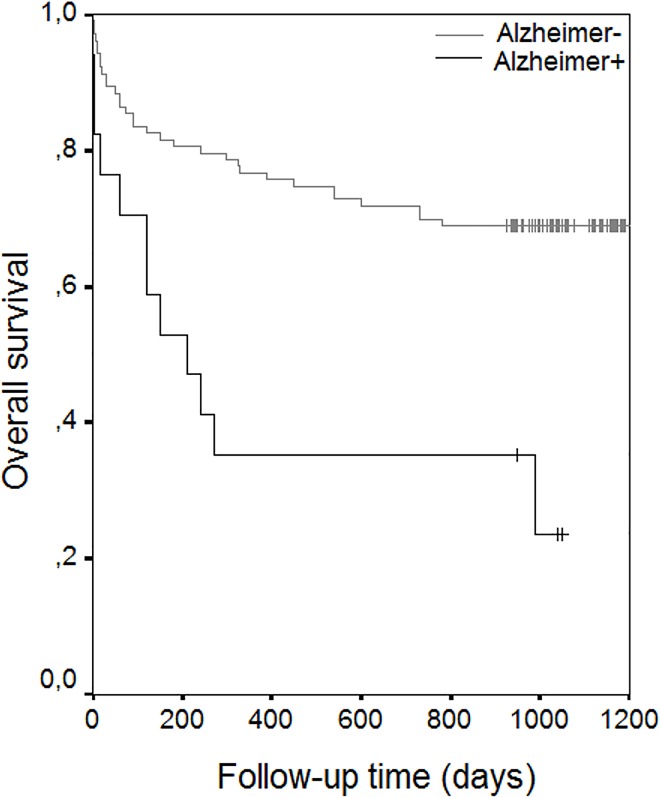
Overall Survival Rate Graphics of Patients with Parkinson’s Disease,

**Fig 4:**
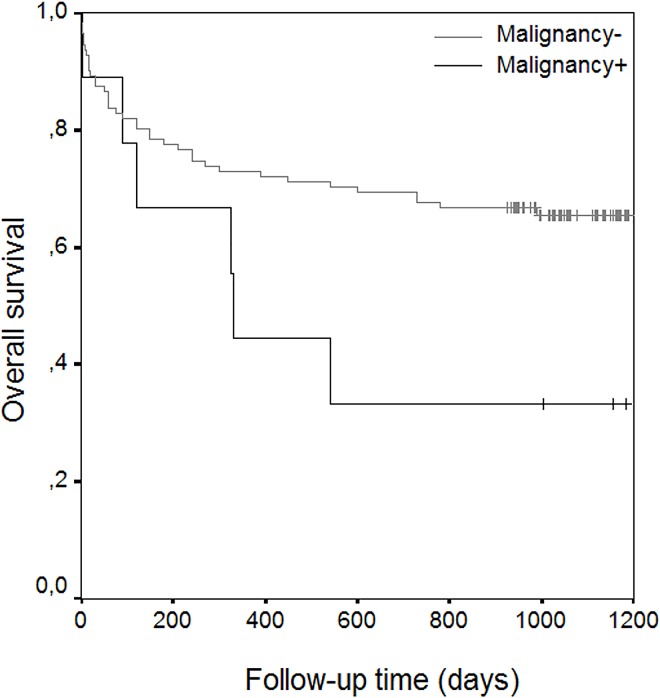
Overall Survival Rate Graphics of Patients with Malignancy.

**Fig 5:**
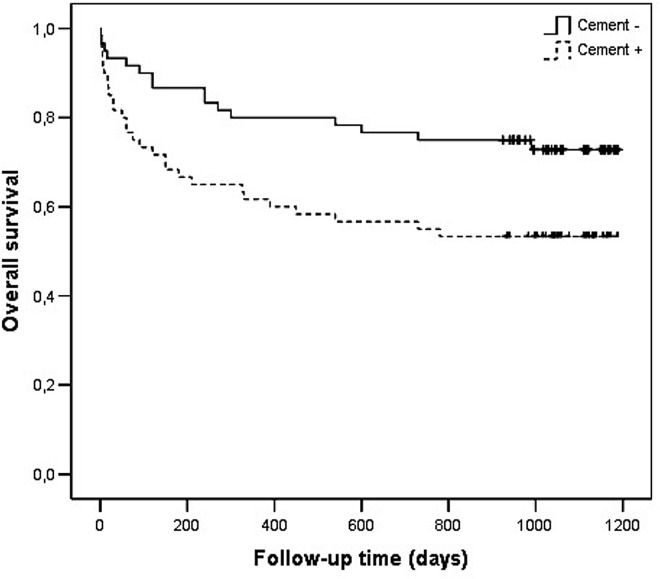
Overall Survival Rate Graphics of Patients with Cementation.

The results of univariate analysis showed statistically significant risk factors to be age (p< 0.001), ASA scores (p< 0.001), associated diseases such as coronary artery disease (p:0.007), atrial fibrillation (p:0.045), congestive heart failure (p< 0.001), Alzheimer’s disease (p<0.001), Parkinson’s disease (p<0.001), and cementation for prosthetic fixation (p<0.020) (**Table 4**) (Figs 2 and 3) (Figs 4 and 5). With increasing ASA scores, mortality increased 3.305 times with each grade. The presence of coronary artery heart disease increased mortality 2.271 times, Alzheimer’s disease 3.206 times and cemented prosthesis use 2.624 times.

Although not a significant risk factor in univariate analysis, malignancy was found to significantly increase mortality by 6.497 times in the multivariate analysis (Table 4), (Fig 4). Two patients had gastric carcinoma, 2 had an intracranial tumor, 2 had carcinoma of prostate, 1 had multipl myeloma, 1 had genitourinary tumor and 1 had a pancreatic tumor.

The causes of deaths were as follows: emboli (n:20, 45.5%), myocardial infarction (n:4;9.1%), sepsis (n:3;6.8%), natural (n:7, 15.9%), pneumonia (n:5; 11.4%), malignancy (n:3;6.8%), and cerebrovascular event (n:2; 4.5%) (**Table 5**)

**Table 5:** Frequency Distribution of Causes of Deaths.

Cause of Death	N:	%
Emboli*	20	45.5
Natural	7	15.9
Pneumonia	5	11.4
Myocardial Infarction	4	9.1
Sepsis	3	6.8
Malignacy	3	6.8
Cerebrovascular Event*	2	4.5
**Sum**	**44**	**100.0**

*Emboli(thrombo-embolic events(pulmonary, cardiac, bone cement implantation syndrome), Cerebrovascular event(thrombotic or ischemic cerebral infarction or hemorrhage)

After univariate analysis, multivariate analysis was applied. Using the Multiple Cox Proportional Hazards Regression Backward LR method, the best predictors of survival and exitus after hip fracture surgery were analyzed. Of all the risk factors, eight statistically significant risk factors were identified as Parkinson’s disease, ASA scores, Alzheimer’s disease, malignancy, congestive heart failure, age, perioperative complications such as hypotension, and cemented prosthesis fixation (**Table 6**). In contrast to the results of the univariate analysis, hypertension, atrial fibrillation and coronary artery heart disease were not determined to have a significant effect on mortality in the multivariate Cox Regression Analysis (Table 4).

**Table 6:** Best Predictors of Overall Survival By Multiple Cox Proportional Hazards Regression Backward LR method.

Variables	Hazard Ratio	95% Confidence Interval	p-value
**Age**	1.052	1.009–1.096	**0.016**
ASA*	2.803	1.539–5.103	**<0.001**
CHF*	2.370	1.203–4.670	**0.013**
**Alzheimer**	3.438	1.551–7.618	**0.002**
**Parkinson**	5.691	1.960–16.522	**<0.001**
**Malignancy**	4.281	1.596–11.479	**0.004**
**Per-op Complication**	2.549	1.175–5.529	**0.018**
**Cementation**	2.135	1.047–4.354	**0.037**

*ASA(American society of Anesthesiologists), CHF(Congestive Heart Failure)

## Discussion

Hip fracture is very common among the elderly population and is generally felt to be a significant adverse event with a poor outcome. In many studies, mortality after hip fractures has been reported as 20–30%.**[3,6,7,12]**.This high mortality rate is due not only to trauma or major surgery but also to co-existingcardiac, pulmonary or renal diseases, old age and preoperative cognitive dysfunction **[3–5, 12–15].** Elderly patients are particularly prone to mortality in the first year after a hip fracture with mortality rate ranging from 14% to 36%**[1,6,7]**.

The aim of the present study was to determine the mortality rate in a 3-year follow-up period and to determine the risk factors of mortality in patients who had undergone hip fracture surgery in our hospital in 2011. The secondary aim of this retrospective analysis was to report the effects of anesthesia techniques and cemented or uncemented prosthesis implantation on mortality.

In epidemiological studies hip fracture has been associated with a significantly increased mortality risk in 6–12 months after the injury. After the first year, the mortality rate has been reported to be similar to that seen in patients without hip fractures in respect of age and gender. In a study by Chariyalertsak, it was reported that male gender, old age, chronic diseases, poor ambulation before fracture and non-operative management were associated with increased mortality **[7,8,16]**. Sepah found an increased incidence of mortality in patients with non-functional ambulation, male gender, cardiovascular disease, and two or more comorbid conditions**[17,18]**. The current study also showed that old age, a high ASA score, comorbidities such as congestive heart failure, Alzheimer’s disease, Parkinson’s disease, malignancy, cementation and the perioperative hypotension significantly increased mortality. In the 3-year period survival analysis, mortality was determined as 29.17% (35/120) in the first year, 33.33% (40/120) in two years and 36.67% (44/120) in three years. These values are consistent with previous results reported in literature. In our study only patients operated for hip fractures in a one year period–only in 2011- were studied.A big sample size of five to ten years period cases would reveal more accurate results. Most previous studies have reported a significantly higher mortality rate in males than females**[16–18].** The Chatterton study had a predominantly female population but mortality was high in males. In our study, the female / male ratio was 1/6 but no significant difference was determined between the genders, which might be due to regional differences in mortality in Turkey compared to other countries.

Cardiovascular disease predisposes patients to the most common and serious postoperative complications. Heart failure and pulmonary infection are considered to be major postoperative complications in elderly patients undergoing surgery for hip fracture**[5].** In a cohort study in Germany, cardiovascular diseases were reported to be the major cause of death after hip fracture**[7]**. However, in England broncho-pneumonia was the most common mortality risk factor of in-hospital deaths**[7]**. Persistent hypoxia may be present in all patients with hip fracture from the time of admission until up to several days postoperativelyand episodes of myocardial ischaemia may occur in postoperative patients with known ischemic heart disease**[19, 20]**.According to previous studies, patients with circulatory disease might be more likely to fall and sustain a hip fracture as a consequence of impaired circulation and this impairment might also increase the risk of death even after the fracture**[21,22].** Therefore patients with heart and circulatory diseases have higher hip fracture rates than the healthy population and have been reported to have a higher mortality risk even without hip fracture than the other diseased people. This might therefore result in an increased mortality rate in hip fracture patients compared to other diseases. In the current study, HT (74%) and CAHD (40%) were found to be the most associated comorbidities in the patients. Consequently, in parallel with literature, the results showed that patients with heart disease, coronary artery heart disease, atrial fibrillation and congestive heart failure had statistically significant higher mortality rates in univariate analysis than the other patients without these diseases**[19–22]**. It was also observed in the current study that circulatory diseases such as emboli(45.5%) (pulmonary, venous or bone cement implantation), myocardial infarction (9.1%) and cerebrovascular events (4.5%) and bronchopneumonia (11.4%) were the leading causes of more than 50% of mortality. These results are supported by those of Chatterton where it was also found that cardiorespiratory diseases were the leading causes of mortality.

Thrombo-embolic events in hip fracture patients, either pulmonary emboli or bone cement implantation syndrome, have been held responsible for mortality in many studies **[7–10]**. In hip fracture patients fatal pulmonary embolism has been reported to be even more common than in electively operated patients for total hip arthroplasty**[10]**. Therefore, it is important to assess patients for venous thromboembolism risk before surgery. This may guide orthopedic wards to be aware of the emboli risk and to take precautions to prevent it. Cement use itself can also lead to an increased embolic load and thus trigger cardiovascular adverse effects causing hypotension and even collapse and death of the patient**[10]**. Cemented implants in the surgical treatment of hip fractures have the advantages of decreased postoperative pain, better early mobilization and decreased early reoperation. However, an increasing number of studies have reported in-hospital mortality due to cemented prosthesis usage**[10]**. The results of the current study also indicate that cemented hip arthroplasty leads to significantly increased mortality when compared with uncemented prosthesis in hip fracture patients. Cement use seems to be a mortality risk factor especially in early postoperative deaths. There are studies that have identified risk factors in the first 24 hours and 48 hours after surgery. In the current study, it was not primarily aimed to assess the very early postoperative in-hospital deaths but it was observed that in the first 7 days, only two patients with uncemented prosthesis died whereas seven patients died when cemented prosthesis was used. It was known that cemented implants were widely used in our hospital (50%) and it was surprising to see that it was especially used in high risk patients with ASA scores of III and IV. Cemented arthroplasty was determined as an important mortality risk factor in the current study. These results have indicated that the use of cemented implants in this subgroup of patients should be ceased and there must be careful consideration when deciding on the necessity of use.

In the current study, 18 patients had the perioperative complication of hypotension. Cemented implant use (bone cement implantation syndrome), type of anesthesia and blood loss during surgery alone or in combinations might have been the cause of hypotension. Although the main reason could not be identified, it was observed that those hypotensive patients had ASA scores of III and IV (15/18) and seven had cemented implants and half of them died in the follow-up period. These results are comparable with the results of White **[10]**.

General anesthesia and spinal anesthesia were the most commonly used anesthesia techniques in this study at 54.2% and 19.2% respectively. However it was surprising that general anesthesia was the most administered type of anesthesia also in patients with ASA scores of III and IV. It was expected that regional anesthesia techniques would be preferred for those high risk patients but the opposite was determined. There have been few studies about the correlation between anesthesia techniques and mortality in hip fracture patients **[7–9]**. Some have reported a decreased mortality after spinal anesthesia whereas others have found no difference between general anesthesia and spinal anesthesia. In the current study, no statistically significant difference was determined between anesthesia techniques. Our small sample size might be a reason that we couldn’t find a correlation between anesthesia type and mortlity. Well designed big sample sized studies are needed to identify the best type of anesthesia for hip fracture patients. New studies about anesthesia modes of general anesthesia and regional techniques, alone or in combinations have to be planned. The effect of anesthesia techniques on perioperative hypotension and hypoxia, pain, cardiovascular and respiratory complications and delirium are also topics that have to be explored to decrease mortality in hip fracture patients.

Those patients should be easily recognized by nurses and doctors on the ward and managed with a multidisciplinary approach, especially with an orthogeriatric team **[10]**. Those patients should also be assessed systematically in the perioperative period by the anesthesiologists in respect of liberal blood transfusion, invasive physiological monitorization for fluid therapy and even anesthetized by senior anesthesiologists to decrease complications **[10]**.

A Greek study of 499 hip fracture patients revealed higher mortality rates in those with a trochanteric fracture compared to those with a femoral neck fracture in a 5 and 10-year follow-up period after the event**[23]**. A Danish study of 2674 hip fracture patients with femoral neck fracture reported that the mortality rates between neck and trochanteric hip fracture patients were not significantly different during a mean follow-up of 2.6 years **[24]**.

In the current study, 87 patients (72.5%) had a femoral neck fracture and the remaining 33 patients (27.5%) had a trochanteric hip fracture. The mortality was 48.5% in trochanteric fracture cases and 32.2% in patients with a femoral neck fracture. The incidence seemed to be higher in the patients with a trochanteric fracture but the type of surgery, type of fracture, whether trochanteric or femoral neck, and the occurrence of refracture were found to have no significant effect on mortality in this study. This result is comparable with the results of similar studies**[16, 19, 20]**.

Yukiharu determined dementia as a risk factor for mortality after hip fracture **[4]**. Previous studies have also reported similar results. Such patients are usually immobile or need care-giver assistance and are more prone to respiratory complications such as aspiration pneumonia**[25]**. In a Norwegian study it was reported that dementia/cognitive impairment were risk factors of mortality from the second postoperative day to a 6-year follow-up period. This was stated to be due to patients with impaired cognitive function patients not being able to follow the guidelines and postoperative procedures such as respiratory physiotherapy and mobilization to enhance recovery after surgery **[8]**. In the current study, Parkinson’s disease and Alzheimer’s disease were found to be significant risk factors for mortality and these results are comparable with the results of other similar studies. Outcomes of mortality after hip fracture might be improved by nutritional supplementation and comprehensive multidisciplinary intervention programs, especially in dementia patients**[26–28]**. Long-term co-operation between primary healthcare and specialist healthcare must be enhanced to improve survival after hip fracture. New studies are needed to find special medical care systems to reduce the incidence and severity of complications after hip fracture surgery and improve the standard care of elderly patients with hip fracture **[13,17]**.

Roche reported the presence of three or more comorbidities as a significant risk factor for mortality after hip fracture **[13]**. The correlation between mortality and ASA grades has also been shown in recent studies **[29,30]**. Increased comorbidity and therefore, high ASA scores, have been found to be the strongest mortality predictor among hip fracture patients**[7]**. The current study results support reports of these recent studies as associated comorbidities were similarly found to play a role in mortality. Of 44 patients who died, 42 had an ASA score of III or IV and mortality was determined to increase 3.305 times with each increase in ASA score. The ASA score is determined by associated diseases and their degree of involvement and reflects the overall physiological state of the patient. Therefore, it is not surprising to see increased mortality with higher ASA scores.

In the current study, mortality was 29.17% (35/120) in the first year, 33.33% (40/120) in two years and 36.67% (44/120) in three years, according to the survival analysis. Gender, type of anesthesia technique, type of surgical technique (cemented vs. uncemented), place of residence before fracture, type of fracture and the occurrence of refracture during the follow-up period were not found to have a statistically significant effect on mortality after hip fractures. Rather, it was found that increasing age, high ASA scores, presence of congestive heart failure, Alzheimer’s disease, Parkinson’s disease, malignancy, perioperative complications such as hypotension and cemented prosthesis fixation were major risk factors of mortality after hip fractures in a 3-year follow-up period.

In conclusion,early detection and treatment of that subgroup of patients with experienced senior practitioners in a multidiciplinary approach, perioperative care including advanced monitorization methods and additionally an awareness of bone cement implantation syndrome and use of uncemented prosthesis and attempts to identify the best type of anesthesia in high risk patients may all improve the outcomes for hip fracture patients.
